# Growth and Morphological Responses of Duckweed to Clonal Fragmentation, Nutrient Availability, and Population Density

**DOI:** 10.3389/fpls.2020.00618

**Published:** 2020-05-25

**Authors:** Li-Min Zhang, Yu Jin, Si-Mei Yao, Ning-Fei Lei, Jin-Song Chen, Qian Zhang, Fei-Hai Yu

**Affiliations:** ^1^Institute of Wetland Ecology & Clone Ecology, Zhejiang Provincial Key Laboratory of Plant Evolutionary Ecology and Conservation, Taizhou University, Taizhou, China; ^2^College of Life Science, Sichuan Normal University, Chengdu, China; ^3^Institute of Environment, Chengdu University of Technology, Chengdu, China

**Keywords:** aquatic plant, clonal plant, disturbance, duckweed, floating plant, *Spirodela polyrhiza*

## Abstract

Connected ramets of aquatic clonal plants are susceptible to fragmentation by disturbance. Such clonal fragmentation may interact with nutrient availability and individual density to affect growth and morphology of aquatic clonal plants. We grew the widespread floating clonal plant *Spirodela polyrhiza* (duckweed) under three levels of population density (low, medium, or high), two levels of nutrient availability (low or high), and two levels of clonal fragmentation (with or without). Clonal fragmentation and high nutrients increased biomass and ramet number, but decreased frond width, frond length, and specific frond area of *S. polyrhiza*. Increasing population density decreased growth (biomass and ramet number) and frond and root size, and increased frond thickness of individual ramets of *S. polyrhiza*. The negative effect of population density on growth of *S*. *polyrhiza* was greater under high than under low nutrient availability. Furthermore, the negative effect of population density on total mass and frond mass of *S. polyrhiza* was greater with fragmentation than without. These results suggest that clonal fragmentation, nutrient availability and population density can interact to affect growth and morphology of clonal floating plants. Competition for nutrients and space, rather than light, may be the mechanisms underlying reduced growth of clonal floating plants. As clonal fragmentation can increase biomass and ramet production of *S*. *polyrhiza*, disturbance that potentially causes clonal fragmentation cannot be recommended as a measure to limit the spread of clonal floating plants.

## Introduction

Small floating plants are conspicuous components of many aquatic ecosystems (Hillman, 1961; Kanoun-Boulé et al., 2009) and play an important role in carbon fixation and nutrient cycling (Padial et al., 2009). Among these small floating plants, duckweeds (Lemnaceae) are often dominant and form dense floating mats in eutrophic aquatic ecosystems such as lakes, ditches, and ponds (Dickinson and Miller, 1998). This is because duckweeds are capable of very fast clonal propagation and their population doubling time is as short as 1–2 days under favorable, nutrient-rich conditions (Ziegler et al., 2015). However, under the floating mat of duckweeds, water often becomes too anoxic for animals to survive (Scheffer et al., 2003) and light becomes too attenuated for submerged macrophytes to coexist (Driever et al., 2005). As a result, the biodiversity of duckweed-dominated, eutrophic systems are usually low (Driever et al., 2005). Thus, understanding mechanisms affecting establishment and persistence of duckweeds is critical for biodiversity conservation and restoration of these aquatic ecosystems.

Aquatic ecosystems are frequently subjected to disturbance caused by e.g., flooding (Voesenek et al., 2004; Huber et al., 2014), human activities (Resh et al., 1988; Lin et al., 2012) and grazing by waterfowl, fish, and other aquatic animals (Lauridsen et al., 1993). For clonal floating plants, organs (e.g., stolons or rhizomes) connecting ramets of the same clone are often little lignified (Barrat-Segretain, 1996). As a result, strong disturbance can easily fragment clones of floating plants (Wang et al., 2014a), resulting in clonal fragments of different sizes (i.e., consisting of different numbers of ramets) (Zhang et al., 2019). Such clonal fragmentation may have played an important role in the adaptation of floating plants to disturbance (Zhang et al., 2019). Thus, examining the impacts of clonal fragmentation on growth and morphology of floating plants such as duckweeds may give insights into their adaptive strategies to cope with disturbance.

Clonal fragmentation has been frequently shown to decrease growth of the whole plant (i.e., clone) due to loss of the ability of sharing resources (e.g., photosynthates, nutrients and/or water) between previously interconnected asexual individuals (i.e., ramets) (Yu et al., 2002; Song et al., 2013; Elgersma et al., 2015; Roiloa and Retuerto, 2016; Wang et al., 2017; Lin et al., 2018; Lu et al., 2020). However, there are also studies showing that clonal fragmentation had no impact on whole plant growth (Wang et al., 2009; Xiao et al., 2011; Roiloa and Retuerto, 2012), including some floating plants such as *Pistia stratiotes* and *Eichhornia crassipes* (Wang et al., 2014a; Li et al., 2015). This is likely because reduced growth of the recipient ramets importing resources (from their connected donor ramets) is compensated by increased growth of the donor ramets exporting resources (Van kleunen et al., 2000; Wang et al., 2009, 2014a; Xiao et al., 2011; Roiloa and Retuerto, 2012). Furthermore, clonal fragmentation of aquatic plants may increase ramet production and thus their potential vegetative dispersal (Wang et al., 2014a).

Population density is an important factor for plant growth and morphology because it affects resource availability (Van kleunen and Fischer, 2003; Wang et al., 2014a). High population density often causes reductions in plant growth (Van kleunen et al., 2001; Driever et al., 2005; Jiang et al., 2010) and changes in morphology such as leaf size and thickness (Coelho et al., 2000; Coll et al., 2004). Compared to land plants or submerged plants, floating plants are less limited by population density as they can freely move in water surface. However, in a particularly limited space such as small ditches and ponds, fast clonal growth and frequently clonal fragmentation may greatly increase population density of floating plants. Under such conditions, population density may become an important factor regulating their growth and morphology.

Nutrient availability is a key factor influencing performance of aquatic plants (Khan and Ansari, 2005). Particularly, the concentrations of nitrogen and phosphorus are thought to control the abundance of floating plants (Szabo et al., 2010; Smith, 2014). Previous work investigated interactions between nutrients and intraspecific competition (Klimeš and Klimešová, 1994; Creed et al., 1997; Steen and Scrosati, 2004), and between population density and clonal fragmentation (Wang et al., 2014a). However, we know little about the complex interactions between all three factors – clonal fragmentation, population density, and nutrient availability. In a previous study, we tested the effect of the interaction between fragment length, interspecific competition and nutrient availability on *Salvinia natans* (Zhang et al., 2019), but the interactive effect of clonal fragmentation (i.e., fragmentation vs. non-fragmentation), population density, and nutrient availability appears not to have been studied so far.

Duckweeds are a food source of a number of vertebrates (Landolt and Kandeler, 1987), so that clonal fragmentation occurs frequently in duckweeds. We selected the widespread, floating, clonal plant *Spirodela polyrhiza* (duckweed), and grew one (low density), four (medium density) or 16 (high density) ramets in containers with two levels of nutrients (low or high). All newly produced ramets were either severed (with fragmentation) or connected to parent ramets (without fragmentation) to test the effects of clonal fragmentation, nutrient availability, population density, and their interactions on growth and morphology of *S. polyrhiza*. Specifically, we addressed two questions (1) Do clonal fragmentation, nutrient availability and population density affect growth and morphology of *S. polyrhiza*? (2) Do clonal fragmentation, nutrient availability and population density interact to affect growth and morphology of *S. polyrhiza*?

## Materials and Methods

### Study Species

*Spirodela polyrhiza* (L.) Schleiden is a small clonal plant of the Lemnaceae family (duckweeds) and is widely distributed across many temperate and tropical regions of the world (Landolt, 1986). Its fronds are flat, obovate, 5–10 mm long and 3–8 mm wide. It grows rapidly, and floats on water surface. Duckweeds are the morphologically simplest flowering plants (Wang et al., 2014b), but *S. polyrhiza* is the morphologically most complex species of duckweeds (Landolt and Kandeler, 1987). This species primarily reproduces vegetatively, producing daughter fronds from parent fronds at the proximal end in two meristematic pocket regions (Hillman, 1961). Each ramet consists of one or two fronds and 5–11 roots. Ramets are connected by a weak stipe. The species preferably inhabits quiet permanent waters such as ditches, shallow pools, and also slow-moving streams (Jacobs, 1947; Stomp, 2005).

Ramets of *S. polyrhiza* were collected on 4 July 2018 from a slow-moving stream in Taizhou (28°3′N, 121°21′E), Zhejiang Province, China. The species was brought to a greenhouse at Taizhou University for propagation, and the plants were washed several times with double distilled water to avoid microbial and other contaminations. They were then rinsed with 0.01 M NaClO for 30 s to prevent algal growth (Wei et al., 2010), and cultivated in tanks (64 cm long × 42 cm wide × 14 cm deep) filled with 20 L of 10% Hoagland solution (Hoagland and Arnon, 1950). The Hoagland solution contained 945 mg/L Ca(NO_3_)_2_⋅4H_2_O, 506 mg/L KNO_3_, 80 mg/L NH_4_NO_3_, 136 mg/L KH_2_PO_4_, 493 mg/L MgSO_4_, 13.9 mg/L FeSO_4_⋅7H_2_O, and 18.7 mg/L EDTA⋅2Na.

### Experimental Design

The experiment had three levels of initial population density (low, medium, or high) crossed with two levels of nutrient availability (low or high) and two levels of fragmentation (with or without fragmentation) in a fully factorial design with seven replicates each. In the low population density treatment, one ramet of *S*. *polyrhiza* was added in a container. In the medium and high population density treatments, four and 16 ramets were added, respectively. Ramets consisting of two fronds were used in this experiment. Each replicate was comprised of a container that was 24.5 cm in diameter and 11 cm tall, filled with 3 L of either a low (2%) or high concentration (10%) of Hoagland solution.

Clonal fragmentation was applied by severing connection between parent ramets and offspring ramets. In half of the containers, we severed stipe connections between the parent ramets and the newly produced offspring ramets; in the other half, the stipe connections were left intact. Severing was repeated every 3 days during the experiment. We randomly selected 200 ramets to measure initial biomass (biomass of 10 ramets of *S. polyrhiza*: 16 ± 0.5 mg, mean ± SE, *n* = 20). Containers were arranged randomly in the greenhouse.

The experiment lasted for 5 weeks, starting on 16 July and ending on 19 August 2018. At harvest, plants in medium and high population density treatments covered the entire water surface. Hoagland solutions were completely replaced every week. Photosynthetic photon flux density at the water surface was measured at noon on sunny days (702–826 μmol m^–2^s^–1^; LI-250A; LI-COR Biosciences). There were about 14 h of daylight and 10 h of darkness in the greenhouse during the experiment. The mean air temperature was 27.0 ± 0.3°C (mean ± SD), and the mean relative humidity was 85.9 ± 0.6% (mean ± SD), as measured hourly by temperature loggers (iButton DS1923; Maxim Integrated Products, United States).

### Measurements

By the end of the experiment, we counted the total ramet number of *S. polyrhiza* in each container. We randomly selected 25 ramets in each container and measured morphological traits, including frond width, frond length, and longest root length (hereafter “root length”) of each ramet. In each container, we obtained an image of 25 randomly selected ramets by a scanner (Epson Perfection V19), and measured frond width, frond length, and total frond area by WinFOLIA Pro 2004a (Regent Instruments, Canada) using this image. These ramets were then separated into fronds and roots, dried in oven at 70°C for 48 h, and weighed to obtain total frond mass and total root mass of these 25 ramets. Specific frond area in each container was calculated as total frond area divided by total frond mass of these 25 ramets, which can be used to reflect frond thickness and light use efficiency of fronds. Mean frond width, frond length, longest root length, mass per ramet and frond area per ramet were also calculated based on the data of the 25 ramets in each container. In each container, we randomly selected another 75 ramets, and separated them also into fronds and roots. Frond mass and root mass of these 75 ramets were obtained after oven-drying at 70°C for 48 h. Based on frond mass and root mass of the 100 ramets (i.e., the sum of the 25 ramets for measuring morphological traits and frond and root mass, and the 75 additional ramets to measure frond and root mass), we obtained frond mass ratio (frond mass/total mass) and root mass ratio (root mass/total mass). The remaining plants in each container were also harvested, but not separated into leaves and roots. They were dried in oven at 70°C for 48 h, and weighed. Total mass in a container was the sum of mass of the 100 ramets and the remaining ones in that container. Total frond mass in a container was calculated as total mass × frond mass ratio in that container, and total root mass was total mass × root mass ratio.

### Data Analysis

Before analysis, values of total mass, frond mass, root mass, and ramet number were divided by the number of parent (initial) ramets (i.e., 1, 4, or 16), so that values were scaled to the level of per parent ramet. We used three-way ANOVA to test for the effects of nutrient availability (low or high), population density (low, medium, or high), and fragmentation (with or without fragmentation) on growth (final ramet number, total mass, frond mass, and root mass) and morphology (frond width, frond length, root length, specific frond area, mass per ramet, and frond area per ramet) of *S*. *polyrhiza*. Ramet number and root length were log-transformed to improve homoscedasticity; figures show untransformed data. Statistical analyses were carried out with SPSS 19.0 (IBM, Armonk, NY, United States).

## Results

### Effects of Clonal Fragmentation, Nutrient Availability, and Population Density on Growth

**FIGURE 1:**
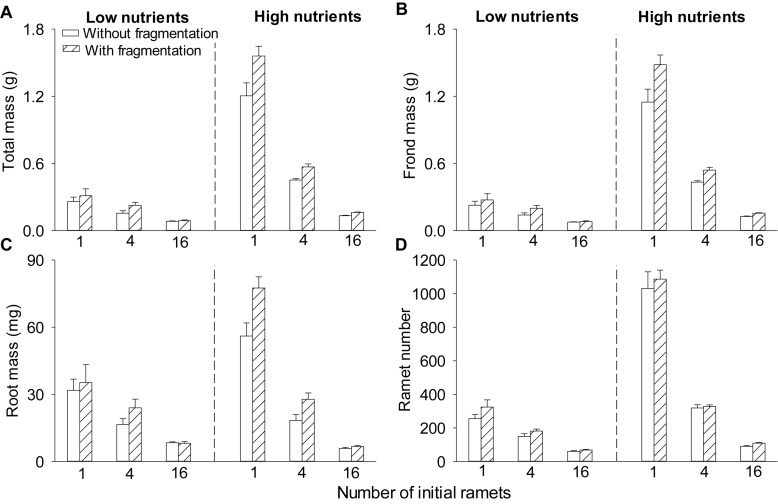
Effects of clonal fragmentation, population density, and nutrient availability on **(A)** total mass, **(B)** frond mass, **(C)** root mass, and **(D)** ramet number of *Spirodela polyrhiza*. Values of mass and ramet number per container were divided by the number of parent (initial) ramets (i.e., 1, 4, or 16). Values are mean + SE (*n* = 7). See Table 1 for ANOVAs.

Clonal fragmentation increased total mass, frond mass, root mass, and ramet number of *S*. *polyrhiza* by 6.0–50.6% compared to the treatment without fragmentation (Table 1 and Figures 1A–D). High nutrient levels increased total mass, frond mass, and ramet number of *S*. *polyrhiza* by 49.8–439.3% compared to low nutrient levels (Figures 1A,B,D). High nutrient levels also increased root mass in low and medium population density by 11.7–119.7%, but decreased root mass by 24.2% in high population density (Figure 1C). The positive effects of clonal fragmentation on total mass and frond mass in *S*. *polyrhiza* were greater under high than under low nutrient conditions (Table 1 and Figures 1A,B).

**TABLE 1:** Statistical analysis of the effects of clonal fragmentation, nutrient availability, and population density on final total mass, frond mass, root mass, and ramet number of *Spirodela polyrhiza.*

**Effect**	***df***	**Total mass**	**Frond mass**	**Root mass**	**Ramet number**
Fragmentation (F)	1,72	13.07**	12.09**	9.08**	8.01**
Nutrients (N)	1,72	289.96***	293.91***	23.61***	262.64***
Density (D)	2,72	212.04***	198.92***	115.60***	464.14***
F × N	1,72	4.75*	4.48*	2.23^ns^	0.71^ns^
F × D	2,72	3.48*	3.25*	2.28^ns^	0.04^ns^
N × D	2,72	115.93***	114.54***	22.36***	26.88***
F × N × D	2,72	2.42^ns^	2.26^ns^	1.40^ns^	0.61^ns^

Values of mass and ramet number per container were divided by the number of parent (initial) ramets (i.e., 1, 4, or 16) before analysis. The given are F values, degree of freedom (df) and significance levels (*** P < 0.001,** P < 0.01,* P < 0.05, and ^*ns*^P ≥ 0.05) of three-way ANOVA.

Increasing population density decreased total mass, frond mass, root mass, and ramet number of *S*. *polyrhiza* by 27.7–91.4% (Table 1 and Figures 1A–D). The negative effects of population density on total mass, frond mass, root mass, and ramet number of *S*. *polyrhiza* were greater under high than under low nutrient levels (Table 1 and Figures 1A,B). The negative effects of population density on total mass and frond mass of *S*. *polyrhiza* were greater with than without fragmentation (Table 1 and Figures 1A,B). There were no significant interaction effects of clonal fragmentation × nutrients × population density on biomass or ramet number of *S*. *polyrhiza* (Table 1 and Figure 1).

### Effects of Clonal Fragmentation, Nutrient Availability, and Population Density on Morphology

**FIGURE 2:**
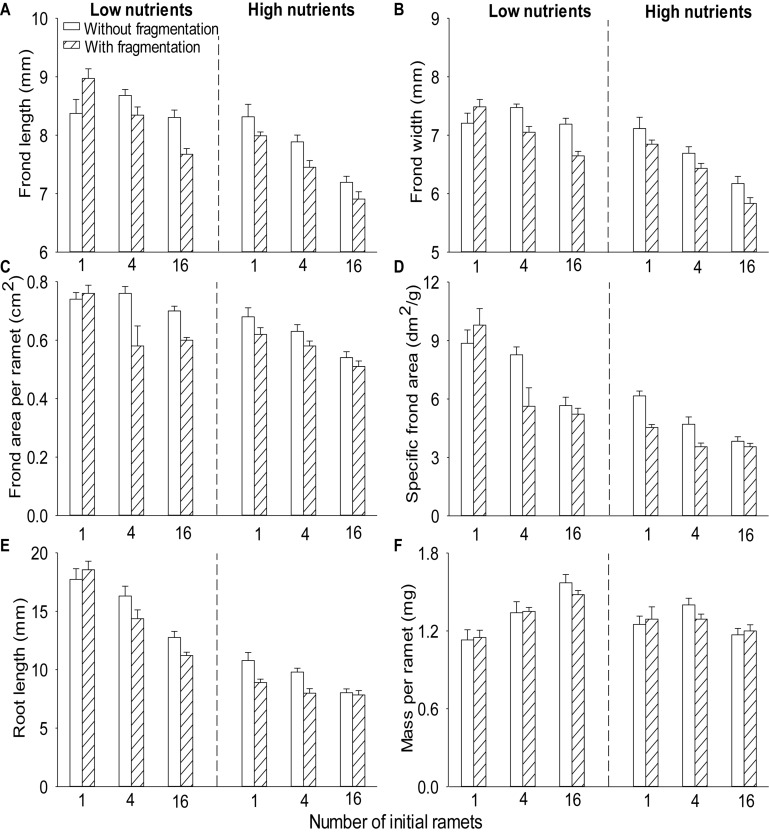
Effects of clonal fragmentation, population density, and nutrient availability on **(A)** frond length, **(B)** frond width, **(C)** frond area per ramet, **(D)** specific frond area, **(E)** root length, and **(F)** mass per ramet of *Spirodela polyrhiza*. Values are mean + SE (*n* = 7). See Table 2 for ANOVAs.

Compared to without fragmentation, clonal fragmentation decreased frond width, frond length, root length and frond area per ramet and specific frond area of *S*. *polyrhiza* (by 3.8–31.9%), except for the treatment of low population density and low nutrient availability (significant main effect of clonal fragmentation and significant clonal fragmentation × nutrients × population density effect; Table 2 and Figures 2A–E). High nutrient levels decreased frond width, frond length, root length and frond area per ramet and specific frond area of *S*. *polyrhiza*, compared to low nutrient levels (Table 2 and Figures 2A–E). Increasing population density significantly decreased frond width, frond length, root length and frond area per ramet and specific frond area of *S*. *polyrhiza* (Table 2 and Figures 2A–E).

**TABLE 2:** Statistical analysis of the effects of clonal fragmentation, nutrient availability, and population density in frond width, frond length, root length, specific frond area, mass per ramet, and frond area per ramet of *Spirodela polyrhiza*.

**Effect**	***df***	**Frond width**	**Frond length**	**Root length**	**Specific frond area**	**Mass per ramet**	**Frond area per ramet**
Fragmentation (F)	1,72	14.84***	7.95**	15.53***	8.68**	0.15^ns^	15.99***
Nutrients (N)	1,72	96.66***	84.39***	398.45***	93.80***	3.92^ns^	32.94***
Density (D)	27,2	36.81***	38.27***	45.93***	20.31***	7.30**	15.45***
F × N	1,72	0.20^ns^	1.85^ns^	1.86^ns^	0.26^ns^	0.04^ns^	1.03^ns^
F × D	2,72	4.08*	5.08**	1.51^ns^	3.10*	0.52^ns^	2.75^ns^
N × D	2,72	5.61**	2.31^ns^	5.13**	4.72*	15.12***	1.46^ns^
F × N × D	2,72	3.36*	4.93*	3.27*	4.19*	0.99^ns^	3.62*

The given are F values, degree of freedom (df) and significance levels (*** *P < 0.001*,** *P < 0.01*,* *P < 0.05, and ^*ns*^P ≥ 0.05) of three-way ANOVA.*

There was no significant interaction effect of clonal fragmentation × nutrients on frond width, frond length, root length, mass per ramet, frond area per ramet, and specific frond area of *S*. *polyrhiza* (Table 2 and Figures 2A–F). The negative effects of plant density on frond width, frond length, and specific frond area of *S*. *polyrhiza* were greater with than without fragmentation (significant clonal fragmentation × population density effect; Table 2 and Figures 2A,B,D). The negative effects of population density on frond width, root length, and specific frond area of *S*. *polyrhiza* were greater under low than under high nutrient levels (significant nutrients × population density effect; Table 2 and Figures 2B,D,E).

## Discussion

### Effects of Clonal Fragmentation, Nutrient Availability, and Population Density on Growth

Not surprisingly, high nutrient availability increased total mass and ramet number of *S*. *polyrhiza*, agreeing with many previous findings (Lowe et al., 2003; Balliu et al., 2007; Xia and Wan, 2008), including those on other floating clonal plants (Zhao et al., 2006; Jampeetong and Brix, 2009; Szabo et al., 2010). Also, as expected, increasing plant density decreased total mass and ramet number of *S*. *polyrhiza*, suggesting the presence of intraspecific competition (Driever et al., 2005; Wang et al., 2005, 2014a; Michelan et al., 2013).

Effects of clonal fragmentation were tested mostly with stoloniferous or rhizomatous clonal plants; the majority of these studies have shown negative effects of clonal fragmentation on growth of the whole clone (Wolfer and Straile, 2012; Song et al., 2013; Glover et al., 2015; Han et al., 2017; Zhou et al., 2017), and a few have shown no effect (Van kleunen et al., 2000; Wang et al., 2009, 2014a; Xiao et al., 2011; Roiloa and Retuerto, 2012). In the present study, however, clonal fragmentation increased growth of the floating plant *S*. *polyrhiza*.

Unlike stoloniferous or rhizomatous clonal plants, *S*. *polyrhiza* lacks horizontally growing stems (i.e., stolons and rhizomes) and newly produced offspring ramets are connected to their parent ramet by slender stipes. Also, the duration of the connection between parent and offspring ramets are much shorter (usually 3–6 days) compared to stoloniferous or rhizomatous clonal plants whose inter-ramet connections can maintain from one growing season (e.g., for many stoloniferous herbs) or several years (e.g., for many rhizomatous grasses) to several decades (e.g., for some species with woody rhizomes). For such reasons, resource sharing mediated by clonal integration may be weak and not favored at a relative longer time (3 days in the current study).

On the other hand, disconnection of parent and offspring ramets may greatly release apical dominance to stimulate outgrowth of dormant adventitious buds so that more offspring ramets can be formed (Wijesinghe and Handel, 1994; Charpentier et al., 1998; Pauliukonis and Gough, 2004). As a result, clonal fragmentation increased final biomass and ramet production of *S*. *polyrhiza*. Another possible reason is that maintaining connections between parent and offspring ramets of *S*. *polyrhiza* imposed great costs (e.g., metabolic and transpiration costs) on the whole clone, which overweighed the benefits of resource sharing (Oborny and Podani, 1996; Wang et al., 2014a). Thus, the growth reduction of the offspring ramets due to the cease of importing resources from the parent ramet is greater than the growth increase of the parent ramet due to the cease of exporting resources (Wang et al., 2014a), resulting in increased growth after clonal fragmentation.

The positive effect of clonal fragmentation on total mass and frond mass of *S*. *polyrhiza* was higher under high than under low nutrient availability and under low and medium than under high population density. These results are consistent with the highly significant main effect of nutrient availability and population density, showing that both low nutrient availability and high population density greatly limited growth of *S*. *polyrhiza*. For instance, *S*. *polyrhiza* produced 0.5–4 times less total mass in low than in high nutrients and 2–8 times less total mass in high than in low population density. Due to such limited growth, the effect of clonal fragmentation became less significant under low nutrient availability and high population density. Also, clonal fragmentation increased population density (ramet number) of *S*. *polyrhiza*, which could potentially intensify intraspecific competition, particular in high population density.

### Effects of Clonal Fragmentation, Nutrient Availability, and Population Density on Morphology

Across other treatments, clonal fragmentation and high nutrients decreased frond and root size of the individual ramets of *S*. *polyrhiza*, as demonstrated by reduced frond width, frond length, root length, and frond area per ramet. However, they increased frond thickness (as shown by reduced specific frond area) so that they had little effect on mass per ramet. Thus, the increased total mass of *S*. *polyrhiza* under high nutrients and clonal fragmentation was solely due to increased ramet production, but not related to individual ramet size or mass.

Increasing population density decreased frond and root size and increased frond thickness of individual ramets of *S. polyrhiza*. An increase in frond thickness is likely because *S. polyrhiza* in unfavorable conditions can accumulate more starch grains (Hillman, 1961; Sree et al., 2015). For terrestrial plants, as well as many emergent plants, reduced light intensity or competition for light due to high population density commonly results in thinner but larger leaves (i.e., increased leaf size and specific leaf area) (Yao et al., 2016; Li et al., 2017; Puglielli et al., 2017). On the other hand, reduced nutrient availability may reduce leaf size and increase leaf thickness of plants (Knops and Reinhart, 2000; Li et al., 2010). Also, increased crowding may also reduce frond size and increase frond thickness of *S*. *polyrhiza* because this floating plant lacks vertical growth and can only spread horizontally (Hillman, 1961). Therefore, for *S*. *polyrhiza*, it is most likely that increasing population density resulted in increased competition for space or nutrients, rather than light.

The negative effects of population density on root length and specific frond area of *S*. *polyrhiza* were higher under low than under high nutrient availability, and the negative effects of population density on frond length and specific frond area were greater when with clonal fragmentation than without. There was also an interactive effect of clonal fragmentation × nutrient availability × population density on frond width, frond length, root length, specific frond area, and frond area per ramet of *S*. *polyrhiza*. This was because the negative effect of population density was greater under low nutrient availability when the plant was fragmented. These results suggest that clonal fragmentation, nutrient availability, and population density can interact to affect morphology of *S*. *polyrhiza*. Clonal fragmentation increased biomass and ramet density of *S*. *polyrhiza*, likely resulting in increased intensity of intraspecific competition, particular under medium and high population density, which may greatly influence the morphological responses of the plants. However, plants under low nutrient availability and low population density did not occupy the whole surface of containers at the end of the experiment so that intensive competition shaping plant morphology may not occur under low population density.

## Conclusion

Clonal fragmentation, nutrient availability, and population density can interact to affect the growth, ramet production, and morphology of clonal floating plants. Competition for nutrients and space, rather than light, may be the mechanisms underlying reduced growth of clonal floating plants. Clonal fragmentation can increase biomass and ramet production of *S*. *polyrhiza*. As disturbance commonly results in clonal fragmentation of *S*. *polyrhiza*, it cannot be recommended as a measure to efficiently control the spread of this clonal floating plant.

## Data Availability Statement

The raw data supporting the conclusions of this article will be made available by the authors, without undue reservation, to any qualified researcher.

## Author Contributions

L-MZ, N-FL, J-SC, and F-HY contributed conception and design of the study. L-MZ, YJ, S-MY, and QZ performed the experiments and collected the data. L-MZ analyzed the data. L-MZ and F-HY wrote the manuscript. All authors contributed to manuscript revision, read and approved the submitted version.

## Conflict of Interest

The authors declare that the research was conducted in the absence of any commercial or financial relationships that could be construed as a potential conflict of interest.
